# General practitioners’ attitudes and practices regarding sick leave certification for patients with depression in Norway – a cross-sectional study

**DOI:** 10.1186/s12913-024-11974-1

**Published:** 2024-12-05

**Authors:** Øystein Hetlevik, Sabine Ruths, Ina Grung, Stein Nilsen, Berit Bringedal

**Affiliations:** 1 Department of Global Public Health and Primary Care, University of Bergen,, Årstadveien 17, Bergen, 5018 Norway; 2 Research Unit for General Practice, NORCE Norwegian Research Centre, Bergen, Norway; 3 Norwegian Labour and Welfare Administration, Nav, Bergen, Norway; 4 Institute for Studies of the Medical Profession, Oslo, Norway

**Keywords:** Depression, General Practice, Sick Leave, Practice Guidelines, Surveys and Questionnaires, Norway

## Abstract

**Background:**

Depression is among the most frequent reasons for sick leave, whereas health authorities recommend a rather strict practice, arguing that work is health-promoting. We aimed to explore GPs’ attitudes and practices regarding sick leave certification for depressed patients.

**Methods:**

A cross-sectional study using the Norwegian Physician Survey (*N* = 1617, 70% response rate) in 2021. The GPs in the panel (*N* = 221) responded to questions about sick leave certification and cooperation with employers and the Norwegian Labour and Welfare Administration (Norwegian acronym: Nav) regarding patients with depression. We used crosstabulation with chi square statistics and logistic regression models to assess differences among GPs.

**Results:**

Among 221 GPs, 62% often/very often perceived patients’ questions for sick leave certification as the main reason for encountering. A total of 46% often/very often considered patients’ expectations inappropriate, with female GPs more frequently than male GPs (36% vs 56%, *p* = 0.005) and younger GPs more frequently than their older counterparts (*p* < 0.001). Although 68% considered sick leave as part of treatment, only 16% often/very often initiated sick leave unless patients raised the question. Sixty-seven percent of GPs reported to often/very often avoid sick listing, if possible, more females than males. GPs who often/very often considered questions for sick leave inappropriate less often considered sick leave as part of treatment (odds ratio (OR): 0.25; 95% CI: 0.13–0.49), and less often report a well-functioning cooperation with Nav (OR:0.37; 95% CI:0.14–0.96). GPs who often/very often considered sick leave as part of treatment more often proposed sick leave for their patients (OR:4.70; 96% CI 1.57–14.01) and reported a less strict approach to sick listing (OR: 40; 95% CI: 0.20–0.79). Ninety-five percent of the GPs rarely/never had direct contact with patients’ employers, whereas 92% often/very often asked patients about their dialogue with the workplace. Eighty-eight percent of the GPs often/very often experienced cooperation with NAV as good, and 87% often/very often felt trusted by them.

**Conclusions:**

Most GPs reported a strict attitude towards sick leave for depression, whereas one-third had a less strict approach. Different perceptions of the appropriateness of sick listing indicate variations in treatment and access to social security benefits.

**Supplementary Information:**

The online version contains supplementary material available at 10.1186/s12913-024-11974-1.

## Background

Depression is a major public health concern with significant impact on quality of life. Depression can also lead to work disability and is one of the most frequent reasons for sick leave in many countries [1], including Norway. In terms of managing depression-related sick leave, several studies have highlighted the potential benefits of staying at work and the usefulness of workplace facilitation and support [2, 3].

In general, long-term sick leave can have negative consequences for mental health and well-being and can lead to a negative cycle of work disability and social isolation [3, 4]. According to the OECD, the proportion of the population outside the workforce is higher in Norway than in other countries. This is considered to be a result of a generous welfare system, where e.g., sick leave is fully compensated from day 1, up to 12 months [5]. The recognition that absence from work due to sick leave or long-term disability is “extraordinarily high” [5] has led to a broad political agreement to change the pattern. Government policy aims to increase the workforce by making work more economically attractive than sick leave and disability benefits, encouraging physicians to be cautious when issuing sick leave certificates, and collaborating with employers’ and employees’ organizations to improve workplace structures to reduce sick leave, promote return to work (RTW), or combine work with graded sick leave [6].

In Norway, a sick leave certificate is required for the absence of more than three consecutive days (eight days in some cases), and certificates are usually issued by general practitioners (GPs) [7]. The Norwegian Directorate of Health provides guidelines for physicians on how to manage sick leave for any condition, including mental illness [8]. The guidelines regarding depression recommend supporting patients’ return to work as soon as possible, considering individual needs, and, if possible, avoiding sick leave for patients with mild to moderate depression.

Considering the large scale of sickness absence in Norway, there appears to be a discrepancy between the authorities’ guidelines and physicians’ sick leave practices. A registry study in Norway revealed that GPs issued a sick leave certificate to 45% of patients with a depression diagnosis or symptoms [9]. Another study demonstrated a decrease in the proportion of sick listed patients between 2009 and 2015, from 58 to 50% during the first 12 months after a new depression diagnosis [10]. However, the majority of these sick leave spells were short, in line with a recent Swedish study show that the majority of persons with mild and moderate mental health problem had very good prognosis regarding future work participation [11].

The role of GPs in managing sick leave can be challenging as it must balance considerations for regulations and patient advocacy [12–16]. Assessing the work capacity of patients with depression may be especially difficult since reduced function is not directly related to the symptom burden presented [17, 18]. On the one hand, GPs are encouraged to support patients to work as much as possible to promote health and prevent long-term work disability. On the other hand, there are situations where sick leave is necessary to promote recovery and prevent further harm [19]. Duration of depression related sick leave is related to multiple factors, including work related issues, societal attitudes towards depression and socioeconomic conditions [20]. Sick leave certification is based on the dialogue between the GP and the patient as a starting point. It is therefore relevant to shed light on GPs’ attitudes and practices in regard to considering appropriateness, proposing or avoiding sick leave and assessing sick leave as part of the treatment plan.

Several stakeholders are expected to collaborate with the GP to facilitate successful RTW. Employers are legally obliged to provide workplace accommodations for sick listed workers and are responsible for supporting the RTW process. When the sick leave lasts for 8 weeks or more, Nav regularly asks the sick listing physician for information about symptoms, function, and treatment, and may overrule the GP’s decision if considered inappropriate. Nav may invite to a dialogue meeting with the patient, employer and GP in sick leave spells with duration above 6 months. Cooperation between NAV practitioners and GPs is important to achieve optimal follow-up for patients. Yet, GPs have expressed a need for better cooperation with the other stakeholders [21, 22]. They have complained about insufficient communication with Nav, e.g., regarding insufficient structures for discussing individual cases, and have reported feeling left alone in efforts to facilitate successful RTW [14, 23].

### Aims

Considering the apparent discrepancy between political goals, health authorities’ recommendations and GPs’ practices, this survey aimed to explore GPs’ attitudes and practices regarding sick leave certification for patients with depression. We further investigated the associations between GP characteristics, reported attitudes and practices and their responses to the different statements, in order to identify patterns in responses.

## Methods

This is a cross-sectional study among GPs based on the Norwegian Physician Survey of 2021 and is a part of “The regular general practitioner scheme: integrated and equitable pathways of depression care, facilitating work participation” (the Norwegian GP-DEP study) [24], which aims to explore depression care within the Norwegian Regular GP scheme.

### Setting

All residents of Norway have access to public health services through the National Insurance Scheme and are entitled to their own GP according to the Regular General Practitioner scheme. Each GP has a fixed list of patients, with an average list size of 1068 in 2020 [25]. On average, 71% of the population has at least one contact per year with a GP [26]. The GP is gatekeeper to specialist health care.

### Study population

The Norwegian Physician Survey is a biennial survey of a representative panel of physicians working in Norway, ongoing since 1994. Its representativeness is secured through comparison with the members of the Norwegian Medical Association, where 94% of all physicians working in Norway are members. The panel is similar to the population of physicians regarding age, gender, and proportions working in primary care, public and private specialist care.

### Questionnaire

A postal and electronic questionnaire was distributed in December 2020, with two reminders at the beginning of 2021. Approximately 50% among the participating physicians gave a postal response, the other half responded electronically. The full questionnaire included questions about demography, working conditions and working hours, priority setting, ethical dilemmas, the work situation during the pandemic, job satisfaction and workload in addition to a separate section about GP depression care.

The section concerning depression care was to be answered by the GPs in the panel only. The questions in this part of the survey were constructed for this study by the research group from the Norwegian GP-DEP study, including the authors of this paper. The questions were designed based on the existing literature in the field, clinical experience, and the objectives of the larger research project [24], seen as elements of face- and content validity. The introductory text to this section was: “The general practitioner’s role in treating patients with depression. This part of the questionnaire is about follow-up of patients with depression and especially about your collaboration with the individual patient and other agencies that provide help to the patient”. The present study is mainly based on 14 statements under the sub-heading “When sick leave (full or partial) and other measures from NAV may be relevant for one of my patients with depression, the following applies:”. Additionally, we used information regarding GPs’ gender, age, and list size.

The GPs were asked to rate each of the statements on a 4-point Likert scale from 1–4, with the alternatives “very often”, “often”, “rarely”, “never” and “does not apply”.

The original Norwegian questionnaire was piloted by five GPs, and their comments were used to improve the face and content validity by improving the phrasing. An English translation of the questions used in this study is presented in Supplementary file 1.

### Statistical analysis

Descriptive statistics were used to examine the distribution of GP and practice characteristics and their responses to statements about depression care.

We then dichotomized the GPs’ responses, grouping “often” and “very often” into one category and “rarely” and “never” into another. We have cross-tabulated GPs’ gender and age with GPs’ responses in the questionnaire to assess possible variations because (i) female GPs have a higher share of female patients and in general female patients are more often sick-listed [27], and (ii) age as proxy for experience. Chi-square statistics was used to test for statistical variation.

To assess internal patterns in GP responses to different items, we used the items regarding appropriateness in a request for sick leave and sick leave as part of treatment as outcomes variables and used logistic regression models to reveal association with other responses. We present odds ratios (OR) with 95% confidence intervals (95%CI) for crude estimate and estimates adjusted for GP gender and age (as a continuous variable).

There was a low rate of missing data (ranging between 0 and 4% per statement and an average of 1.5% for all statements), and consequently, the risk of attrition bias was very small; therefore, we did not perform any attrition analysis.

The significance level was set to *p* < 0.05. Stata/SE version 18.0 (Stata Statistical Software) was used for the statistical analyses.

### Ethics approval

The survey was approved by the Norwegian Agency for Shared Services in Education and Research. The Regional Committee for Medical and Health Research Ethics found that this study did not require approval from the decision IRB 0000 1870. All the members of the panel provided written consent to participate in the study.

## Results

The survey was sent to the panel of the Norwegian Physician Survey (*N* = 2316), and 1617 completed questionnaires were returned (70% response rate). However, we do not know how many GPs were in the panel since the current work position is not known beforehand but provided by the respondents when completing the survey. Therefore, we cannot calculate the response rate for the subsample of GPs.

Among the 221 GPs who answered one or more questions regarding depression care, we found an equal gender distribution (men 50.0%, women 50.0%), with a mean age of 47.1 years (SD 12.3); 37.3% of the GPs were aged < 40 years, 23.2% 40–49, 12.7% 50–59, and 26.8% > 60 years. The mean list size was 1038 (SD 352).

The statements in the questionnaire used in this study are shown with GPs’ ratings in Table 1 and the gender and age distributions of their dichotomized responses in Table 2.

**Table 1:** GPs’ attitudes and practices regarding sick leave certification for patients with depression (*N* = 221)

		**GPs’ rating of statements:**							
*When sick leave (full or partial) and other measures from NAV may be relevant for one of my patients with depression, the following applies:*	**Number completed**	**Very often**		**Often**		**Rarely**		**Never**	
	*N*	*N*	%	*N*	%	*N*	%	*N*	%
**Items related to GP’s contacts with the patients**									
Questions about sick leave are the main reason why the patient consults me	216	16	7.4	117	54.2	80	37.0	3	1.4
The patient expects sick leave in situations where I do not consider it appropriate	216	13	6.0	86	39.8	116	53.7	1	0.5
I consider sick leave to be part of the treatment	215	7	3.3	140	65.1	65	30.2	3	1.4
I propose sick leave without the patient having raised the issue with me	216	1	0.5	34	15.7	164	75.9	17	7.9
I avoid sick leave as far as possible	217	18	8.3	127	58.5	70	32.3	2	0.9
**Items related to GPs’ contact with employers**									
I engage in dialogue with the patient’s employer without the involvement of NAV	214	0	0	11	5.1	125	58.4	78	36.4
I ask the patient about the dialogue with the employer	215	80	37.5	117	54.4	15	7.0	3	1.4
**Items related to GPs’ contact with NAV**									
NAV seeks advice from me regarding follow-up of the patient	215	3	1.4	71	33.0	108	50.2	33	15.3
NAV considers me an important caregiver for the patient	211	33	15.6	125	59.2	48	22.7	5	2.4
NAV expects other specialists to be involved in longer sick leave	212	60	28.3	92	43.4	56	26.4	4	1.9
NAV trusts my professional assessments	211	45	21.3	140	66.4	24	11.4	2	0.9
The cooperation with NAV works well	214	47	22.0	142	46.7	25	11.7	0	0
I consider myself a coordinator between NAV, the patient, and me	212	36	17.0	99	46.7	64	30.2	13	6.1
NAV considers me a coordinator between NAV, the patient, and me	209	24	11.5	114	54.5	64	30.6	7	3.3

**Table 2:** GPs’ attitudes and practices regarding sick leave certification for patients with depression, by GP gender and age groups (*N* = 221)

**When sick leave (in whole or in part) and other measures from NAV may be relevant for one of my patients with depression, the following applies:**	**Gender**^**a**^							**Age groups**^**b**^								
	**All**		**Male**		**Female**			**Under 40**		**40–49**		**50–59**		**60 + **		
	*N*	%	*N*	%	*N*	%	*p* value*	*N*	%	*N*	%	*N*	%	*N*	%	*p* value*
	221	100	110	50	110	50		82	37.3	51	23.2	28	12.7	59	26.8	
Questions about sick leave are the primary reason why the patient comes to me							**0.010**									0.190
Often/very often	133	61.9	**57**	**53.3**	**76**	**70.4**		54	67.5	34	66.7	15	53.6	29	51.8	
Rarely/never	82	38.1	**50**	**46.7**	**32**	**29.6**		26	32.5	17	33.3	13	46.4	27	48.2	
The patient expects sick leave in situations where I do not consider it appropriate							**0.005**									** < 0.001**
Often/very often	99	46.0	**39**	**36.4**	**60**	**55.6**		**50**	**63.3**	**24**	**47.1**	**13**	**46.4**	**12**	**21.1**	
Rarely/never	116	54.0	**68**	**63.6**	**48**	**44.4**		**29**	**36.7**	**27**	**52.9**	**15**	**53.6**	**45**	**78.9**	
I consider sick leave as part of the treatment							0.330									0.839
Often/very often	146	68.2	69	65.1	77	71.3		54	68.4	37	72.5	19	67.9	36	64.3	
Rarely/never	68	31.8	37	34.9	31	28.7		25	31.6	14	27.5	9	32.1	20	35.7	
I propose sick leave without the patient having raised the issue with me							0.186									0.475
Often/very often	35	16.3	21	19.6	14	13.0		10	12.7	7	13.7	6	21.4	12	21.1	
Rarely/never	180	83.7	86	80.4	94	87.0		69	87.3	44	86.3	22	78.6	45	78.9	
I avoid sick leave as far as possible							**0.021**									0.106
Often/very often	144	66.7	**64**	**59.3**	**80**	**74.1**		50	63.3	35	68.6	24	85.7	35	60.3	
Rarely/never	72	33.3	**44**	**40.7**	**28**	**25.9**		29	36.7	16	31.4	4	14.3	23	39.7	
I engage in dialogue with the patient’s employer without the involvement of NAV							0.769									0.371
Often/very often	11	5.2	5	4.7	6	5.6		3	3.8	1	2.0	2	7.4	5	8.8	
Rarely/never	202	94.8	101	95.3	101	94.4		75	96.2	50	98.0	25	92.6	52	91.2	
I ask the patient about the dialogue with the employer							0.325									**0.007**
Often/very often	196	91.6	96	89.7	100	93.5		**74**	**94.9**	**49**	**98.0**	**26**	**92.9**	**47**	**81.0**	
Rarely/never	18	8.4	11	10.3	7	6.5		**4**	**5.1**	**1**	**2.0**	**2**	**7.1**	**11**	**19.0**	
NAV seeks advice from me regarding follow-up of the patient							0.212									0.692
Often/very often	74	34.6	41	38.7	33	30.6		23	29.5	19	37.3	11	39.3	21	36.8	
Rarely/never	140	65.4	65	61.3	75	69.4		55	70.5	32	62.7	17	60.7	36	63.2	
NAV considers me an important caregiver for the patient							0.177									0.131
Often/very often	157	74.8	82	78.8	75	70.8		53	69.7	34	68.0	24	85.7	46	82.1	
Rarely/never	53	25.2	22	21.2	31	29.2		23	30.3	16	32.0	4	14.3	10	17.9	
NAV trusts my professional assessments							0.675									0.258
Often/very often	184	87.6	93	88.6	91	86.7		64	85.3	41	82.0	26	92.9	53	93.0	
Rarely/never	26	12.4	12	11.4	14	13.3		11	14.7	9	18.0	2	7.1	4	7.0	
The cooperation with NAV works well							0.539									0.588
Often/very often	188	88.3	95	89.6	93	86.9		65	84.4	45	90.0	26	92.9	52	89.7	
Rarely/never	25	11.7	11	10.4	14	13.1		12	15.6	5	10.0	2	7.1	6	10.3	
I consider myself a coordinator between NAV, the patient, and me							0.374									**0.004**
Often/very often	135	64.0	69	67.0	66	61.1		**43**	**55.8**	**26**	**51.0**	**20**	**74.1**	**45**	**80.4**	
Rarely/never	76	36.0	34	33.0	42	38.9		**34**	**44.2**	**25**	**49.0**	**7**	**25.9**	**11**	**19.6**	
NAV considers me a coordinator between NAV, the patient, and me							0.241									**0.010**
Often/very often	138	66.3	71	70.3	67	62.6		**46**	**60.5**	**27**	**54.0**	**19**	**73.1**	**46**	**82.1**	
Rarely/never	70	33.7	30	29.7	40	37.4		**30**	**39.5**	**23**	**46.0**	**7**	**26.9**	**10**	**17.9**	

^a^missing gender, *n* = 1

^b^missing age, *n* = 1

^*^p value estimated by Chi square test

More female than male GPs reported that patient questions about sick leave certification often/very often were the main reason for the encounter (70.4% vs. 53.3%, *p* = 0.01) (Table 2). Furthermore, 55.6% of female GPs considered requests for sick leave often/very often inappropriate, whereas 36.4% of male GPs did (*p* = 0.005). The perception of inappropriateness decreased significantly with increasing age, from 63.3% for GPs under 40 years to 21.1% for those 60 years or older (*p* < 0.001).

While most GPs (68.2%) considered sick leave often/very often to be part of the treatment, 83.8% responded that they rarely/never suggested sick leave without the patient having raised this issue. There was gender variation in the ability to avoid sick leave, if possible, with 74.1% of female GPs responding often/very often to this approach, whereas 59.3% of male GPs did.

Almost all GPs (91.6%) often/very often asked their patients about their dialogue with their employer, but 94.8% of the GPs rarely/never had direct contact with their patients´ employer.

Most GPs (88.3%) rated their cooperation with Nav often/very often as good; 74.8% believed that NAV saw them as important caregivers, and 86.7% regarded Nav as trusting their judgments. However, most GPs (65.4%) rarely/never experienced Nav seeking advice from them. Compared with younger GPs, GPs above 50 years more often saw themselves as coordinators when Nav was involved.

We found no significant associations between GPs’ attitudes and practice and the number of patients on their list (not tabulated).

When assessing the internal association between the GP responses to the items related to patients and Nav, we found a positive association between often/very often considering a request for sick leave as inappropriate and seldom/never considering sick leave as part of treatment, and seldom /never consider cooperation with Nav as well-functioning (Table 3). Further, GPs who often/very often considered sick leave as part of treatment more often reported a less strict attitude regarding avoiding sick leave.

**Table 3:** Associations between GP responses to the items about appropriateness of expectations of sick leave and sick leave as part of treatment, and the other statements regarding their patients with depression, contact with NAV, and GP characteristics

	**The patient o*ften/very often* expects sick leave in situations where I do not consider it appropriate**				**I o*ften/very often* consider sick leave as part of the treatment**			
**GP responses items related to contacts with the patients**	Crude^a^		Adjusted^a^		Crude^a^		Adjusted^a^	
	OR	95% CI	OR	95% CI	OR	95% CI	OR	95% CI
Questions about sick leave are *often/very often* the primary reason why the patient comes to me^b^	**5.00**	**2.69–9.30**	**4.70**	**2.45–9.05**	1.32	0.73–2.37	1.26	0.69–2.30
The patient *often/very often* expects sick leave in situations where I do not consider it appropriate^b^	–	–			**0.33**	**0.18–0.59**	**0.25**	**0.13–0.49**
I *often/very often* consider sick leave as part of the treatment^b^	**0.33**	**0.18–0.59**	**0.25**	**0.13–0.49**	–	–	–	–
I *often/very often* propose sick leave without the patient having raised the issue with me^b^	0.56	0.26–1.19	0.66	0.30–1.46	**4.28**	**1.45–12.66**	**4.70**	**1.57–14.06**
I *often/very often* avoid sick leave as far as possible^b^	1.53	0.86–2.72	1.49	0.80–2.78	**0.45**	**0.23–0.87**	**0.40**	**0.20–0.79**
**GP responses—items related to contact with NAV**								
NAV *often/very often* considers me an important caregiver for the patient^b^	**0.45**	**0.24–0.84**	0.53	0.27–1.05	1.52	0.79–2.93	1.60	0.02–3.12
NAV *often/very often* trusts my professional assessments^b^	0.58	0.25–1.32	0.69	0.29–1.67	0.65	0.25–1.72	0.66	0.25–1.76
The cooperation with NAV *often/very often* works well^b^	**0.36**	**0.15–0.87**	**0.37**	**0.14–0.96**	0.71	0.27–1.88	0.72	0.27–1.93
**GP characteristics**								
Gender (ref = male)	**2.17**	**1.26–3.77**	1.62	0.90-2.89	1.33	0.75–2.37	1.31	0.72-2.39
Age, as continuous variable	**0.95**	**0.93–0.97**	**0.60**	**0.46-0.77**	1.00	0.97–1.02	0.96	1.21-3.49

^a^Logistic regression, crude and adjusted for GP age and gender. Significant associations in bold

^b^Reference value: rarely/never

## Discussion

In this survey of 221 GPs in the Norwegian Physician Survey, we found that two out of three GPs, more female than male GPs, tried to avoid sick listings as much as possible for patients with depression. Although two-thirds considered sick leave to be part of the treatment, very few initiated sick leave unless the patient asked for it. Nearly half of the GPs considered patients’ expectations for sick leave often/very often as inappropriate, with female and younger GPs more likely than their male and older counterparts to do so. Very few GPs had direct contact with patients’ employers, but almost all GPs communicated with patients about workplace conditions. Furthermore, the majority of GPs stated that cooperation with Nav was good and that they felt accepted and respected as professionals by the Nav officers.

A strength of this study is that the panel of physicians in the Norwegian Physician Survey is representative of Norwegian physicians [28]. Also, in the subsample of GPs, we found the distribution of age and list length to be in line with the national sample of GPs, but with a somewhat larger proportion of female GPs (50% vs 45% in the national sample) and employed GPs (19% vs 15% in the national sample). There may still be bias regarding the GPs’ willingness to participate in the panel, and the relatively small number of participants limits external validity. The results are transferable to countries where the GP has a similar role in certifying and following up sickness absence leave, and the general view of the role of work participation in depression care may be valid in an even larger context.

A limitation in this study is that we used depression as a broad term without specifying diagnostic criteria or severity. Depression is generally diagnosed and managed by GPs ranging it from mild to severe [29]. We therefore assumed that the GPs had patients with varying severities of depression in mind when they assessed the statements, leading to a possible response bias. Furthermore, the statements did not distinguish between full or partial sick leave. The GPs’ understanding of these terms may have influenced their ratings, but the consequence for the results is uncertain. Another limitation is the lack of data regarding differences in socioeconomic status in list populations and GPs’ workload, which are factors that may impact GPs’ practices and partly explain variations.

It is reasonable to argue that the Norwegian national guidelines recommend a strict sickness certification practice in regard to mild and moderate depression [8]. Most GPs reported often/very often seek to avoid sick leave certification, as far as possible, but one-third responded rarely/never to have this approach. A Belgian study of GP-diagnosed depression revealed that 31%, 50%, and 19% of the patients had mild, moderate, and severe depression, respectively [29]. Assuming a similar distribution of the severity of depression among patients in Norwegian general practice, the GPs’ responses seem inconsistent with their actual sick-listing practice, as 40–50% of patients with depression are issued a sick leave certificate by their GP [9, 10, 30]. Studies have also shown that most depression-related sick listings are of short duration; 50% of the sick-listed patients returned to work within two weeks, and 70–75% returned to work within three months [9, 30]. Thus, we can assume that the GPs’ responses mostly concerned mild or moderate depression and shorter sick leave spells, where still sick leave can be justified owing to functional impairment.

In addition to the authorities’ overall goal of reducing sickness absences for economic reasons, another aim is that work participation is perceived to be beneficial for mental health [2, 3, 8]. However, a German study revealed no differences in clinical improvement between patients on sick leave and those not on sick leave, indicating that work participation as part of a treatment plan for depression can be questioned, especially regarding shorter periods up to eight weeks [31].

Norwegian Health Authorities’ restrictive recommendation regarding sick listing for depression is more explicit than the guidelines for other health conditions, although it is an underlying principle in all cases where sick leave is an option. The recommendation “to avoid sick leave as far as possible” for mild and moderate depression may have unintended consequences, especially for the patients with depression, where part of the condition can be lack of self-advocacy when describing their problem.

A study regarding patients with unexplained health problems showed that Norwegian GPs had a conscious approach to negotiating sick leave [14]. Furthermore, the frequent use of short-term sick leave documented by registry studies indicates that GPs in practice also use this negotiation approach for patients with depression and try to balance patients’ need for sick leave with the aim of avoiding long-term absence. However, our findings suggest that some GPs perceive this negotiation approach as not well accepted in regard to depression. Other GPs seem to accept the patient´s own evaluation of their situation and accept sick leave in the beginning, using sick leave as part of treatment, but focusing on early RTW. GPs are uniquely positioned to integrate both personal and structural aspects in their patient care decisions and certification of sick leave is not necessarily a sign not fulfilling a gate keeping role. This indicates that the recommendation should be revised to make a clearer distinction between short and longer sick listing spells, in order to avoid inequality in practice.

In the present study, 62% of the GPs reported that questions about sick leave often/very often was seen as inappropriate, strongly associated with perceiving the request for sick leave as the main reason for contact. Depression involves reduced functioning, and functional loss increases with increasing severity [32]. Cognitive impairment and low energy seem to be the main reasons for reduced work capacity [17, 18, 32]. Such symptoms can be difficult for the patient to express clearly, as well as for the GP to capture. We were unable to identify whether the perceptions of appropriateness were based on loyalty to the recommendations to avoid sick leave or on an in-depth exploration of the medical problem underlying the patient’s wish for sick leave, or both. It is possible that GPs who often consider requests for sick leave inappropriate also reported that sick leave was the main reason for encounters. If so, the GP’s perception of appropriateness may influence the content in communication, resulting in a sick leave discussion as the main issue. Furthermore, the association between perceiving sick leave as inappropriate, not seeing sick leave as part of treatment and rarely/never see cooperation with Nav as valuable may indicate variations in GPs’ attitudes that can lead to unequal access to this social security benefit. These findings warrant further research to reveal the background for the different attitudes and experiences regarding sick listing for depression.

The perception of inappropriateness for requesting a sick leave was more common among female GPs and younger GPs. The age difference can be explained by greater familiarity with their patients, better ability to capture the underlying functional problems, or less compliance with guidelines and stronger faith in their own judgement among older GPs. More male GPs reported rarely/never avoiding sick leave. Similar variations in adherence to guidelines have been demonstrated for the treatment of diabetes, where younger GPs and female GPs were more compliant [33]. GP age and gender variation in sick leave practices for patients with depression warrant further research to explore the underlying causes and mechanisms. However, variations in sick leave certification practices suggest that assessing the need for sick leave is a complex task, such as a puzzle [34].

The GPs in the present study had surprisingly different views on sick leave as part of the treatment; every third rarely/never considered sick leave as a therapeutic measure. There may be different interpretations of “part of treatment”, but sick leave as a relief of pressure and to avoid the negative impact of impaired function on work performance can reasonably be considered part of the treatment plan [13]. More than 80% of the GPs rarely/never took the initiative to propose sick leave, a finding that partly contrasts with the fact that two-thirds of GPs see sick leave as part of the treatment. It seems that patients are in the lead in regard to choosing this measure. We found an association between reluctance to initiate sick leave and not considering sick leave as part of treatment. There may be different attitudes among GPs, and the implicit tone of the guidelines may influence a restrictive approach. One may speculate that the GPs hope to avoid sick listing by simply not raising the question.

In recent decades, there has been an increased awareness of the role of the workplace in reducing sick leave and helping patients stay at work despite health problems [35, 36]. GPs are obliged to attend meetings with employers and patients at an early stage in a case of sick listing if one of the parties considers it to be useful [8]. Additionally, GPs are encouraged to seek knowledge about their sick listed patient’s workplace situation. In the present study, only a few GPs communicated with their patients’ employers directly. However, nearly all the GPs talked with the patients about their dialogue with their employer and were thus informed from the patients’ point of view. This approach is in line with the patient-centred approach used in general practice, which gives patients the opportunity to take care of their own situation. This can also be seen as a self-initiated clarification of the GP’s role, as it can be difficult to maintain confidentiality in contact with the employer, especially if workplace-related issues are part of the problem [37]. Improvement of workplace facilitations for patients with depression is probably better taken care of by occupational health services or Nav.

Given the high frequency of sick leave when a depression diagnosis is used, one should expect somewhat more friction with the authorities who approve the sick leave certification from the GP, but our findings indicate a respectful attitude towards the GPs’ assessments from Nav.

Most GPs felt that Nav acknowledged their role as therapists. Nav has no specific requirements for when a GP should refer a patient with depression to specialist care but will sometimes suggest a referral in cases of sick listing lasting more than three months.

GPs in the oldest age group often considered themselves as coordinators between patients and Nav, and many thought that Nav agreed with this, which may reflect changes in the role of GPs during recent decades.

Norwegian policies regarding sick leave and other social security benefits are based on a broad political agreement to keep employment rates high and absence rates low. Sick leave rates are high compared to similar countries, such as Sweden and Denmark, and may justify relatively strict sick leave recommendations. The total employment rate in Norway is, however, also higher than elsewhere [38]. Furthermore, when comparing the percentage of the population outside the workforce in terms of long-term welfare benefits, Norway has the lowest rates of European countries and the US [38]. When employment rates are high, sick leave is expected to follow, since more employees with reduced functional and working capacity are included. Additionally, the low number of people receiving long-term welfare benefits may suggest that the practice of more liberal short-term sick leave may keep individuals in the workforce. This reasoning can support the practice regarding sick leave for depression, as seen in Norway, where most sick leaves last less than three months.

## Conclusions

Most GPs reported acting in line with the recommendations and avoiding sick leave as much as possible for patients with depression. However, one in three GPs reported a less strict approach, possibly due to a clinical judgement that sick leave is warranted in many cases. Different perceptions of the appropriateness of sick leave may indicate different attitudes and variations in treatment for depressed patients, resulting in unequal access to social security benefits. Further research is required to examine the reasons and the consequences of the variation in GPs’ attitudes and practices regarding sick listing for depression.

## Supplementary Information

Supplementary Material 1.

## Data Availability

The data used in this study are a property of the research group and may be accessible upon reasonable request.

## Abbreviations

GP: General practitioner
Nav: Norwegian Labour and Welfare Administration
RTW: Return to work
