# Supplementary material for: General practitioners’ attitudes and practices regarding sick leave certification for patients with depression in Norway – a cross-sectional study
Source: BMC Health Serv Res. 2024 Dec 5;24:1550. doi: 10.1186/s12913-024-11974-1 (PMC11619211; doi:10.1186/s12913-024-11974-1)
Supplement: Supplementary file 1 — Supplementary Material 1. [file 12913_2024_11974_MOESM1_ESM.pdf]

Supplementary fil 1

Sick leave certification for patients with depression – a survey among general practitioners in Norway

The questionnaire used in the Norwegian Physician Survey in 2021 was design for that purpose and included sections regarding:

- A Working conditions and working hours
- B Prioritization
- C Working situation and infection control during the Covid-19 pandemic
- D The GP's role in treating patients with depression
- E Satisfaction, health and workload
- F Background information

The questions in section D were only for the GPs in the panel and this section was introduced with the text:

"D01 to D07 are ONLY ANSWERED BY GENERAL PRACTITIONERS. If you are not a general practitioner, proceed to question E01 on page 12.

D The general practitioner's role in treating patients with depression

This part of the questionnaire is about follow-up of patients with depression and especially about your collaboration with the individual patient and other agencies that provide help to the patient."

In the present study we used only section D02 from the GP part of survey, concerning the GP's experiences with sick leave for patients with depression and their collaboration with employers and the Norwegian Labour and Welfare Administration (NAV). This section is presented in an English translation below. (The questions are somewhat regrouped in the presentation in the paper)

D02	**When sickness absence (full or partial) and other measures from NAV may be relevant for one of my patients with depression, the following applies**	Very often	Often	Seldom	Never	Not Applicable
D02.01	Questions about sick leave is the main reason why the patient consults me	1	2	3	4	9
D02.02	The patient expects sick leave in situations where I do not consider it appropriate	1	2	3	4	9
D02.03	I consider sick leave to be part of the treatment	1	2	3	4	9
D02.04	I propose sick leave without the patient having raised the issue with me	1	2	3	4	9
D02.05	NAV seeks advice from me regarding follow-up of the patient	1	2	3	4	9
D02.06	I engage in dialogue with the patient's employer without the involvement of NAV	1	2	3	4	9
D02.07	I avoid sick leave as far as possible	1	2	3	4	9
D02.08	I ask the patient about the dialogue with the employer	1	2	3	4	9
D02.09	NAV considers me an important caregiver for the patient	1	2	3	4	9
D02.10	NAV expects other specialists to be involved in longer sick leave	1	2	3	4	9
D02.11	NAV trusts my professional assessments	1	2	3	4	9
D02.12	The cooperation with NAV works well	1	2	3	4	9
D02.13	I consider myself as the coordinator between NAV, the patient, and me	1	2	3	4	9
D02.14	NAV considers me a coordinator between NAV, the patient, and me	1	2	3	4	9
